# Operando Modeling of Multicomponent Reactive Solutions in Homogeneous Catalysis: from Non‐standard Free Energies to Reaction Network Control

**DOI:** 10.1002/cctc.201901911

**Published:** 2019-12-11

**Authors:** Pavel O. Kuliaev, Evgeny A. Pidko

**Affiliations:** ^1^ TheoMAT Group ITMO University Lomonosova 9 St. Petersburg 191002 Russia; ^2^ Inorganic Systems Engineering Group Department of Chemical Engineering Delft University of Technology Van der Maasweg 9 Delft 2629 HZ The Netherlands

**Keywords:** Liquid phase thermodynamic analysis, Operando modeling, Homogeneous catalysis, Reaction network, Catalyst deactivation

## Abstract

Optimization and execution of chemical reactions are to a large extend based on experience and chemical intuition of a chemist. The chemical intuition is rooted in the phenomenological Le Chatelier's principle that teaches us how to shift equilibrium by manipulating the reaction conditions. To access the underlying thermodynamic parameters and their condition‐dependencies from the first principles is a challenge. Here, we present a theoretical approach to model non‐standard free energies for a complex catalytic CO_2_ hydrogenation system under operando conditions and identify the condition spaces where catalyst deactivation can potentially be suppressed. Investigation of the non‐standard reaction free energy dependencies allows rationalizing the experimentally observed activity patterns and provides a practical approach to optimization of the reaction paths in complex multicomponent reactive catalytic systems.

## Graphical abstract

**Operando modeling**: Optimization and execution of chemical reactions are to a large extend based on experience and chemical intuition of a chemist. Herewith we present a practical computational approach to investigate condition‐dependencies of reaction free energies in reactive multicomponent multiphase solutions. This method can be readily used to rationally select starting parameters for experimental catalyst tests.

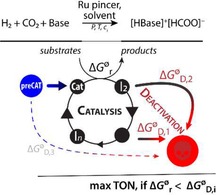

## 1 Introduction

Catalysis plays a pivotal role in the development of new sustainable and energy‐efficient chemical conversion technologies. Although traditionally conversion and catalyst development has been the domain of experimental investigations, the input from the computational modeling has been steadily increasing since the 1980s. The benefits of the synergy between theory and experiment to deliver molecular level insights into complex chemical transformations have become apparent during the last two decades.^1^, ^2^, ^3^, ^4^, ^5^, ^6^ The development of the predictive theoretical approaches to enable a computer‐based search for the optimal conditions and composition of the reaction mixture for a given chemical transformation to replace tedious and costly experimental work is one of the key challenges for applied computational chemistry today.

Chemical reactions in homogenous liquid media are not only controlled by the intrinsic chemistry of the involved components but also by the nature of solvents and conditions at which the reactions are carried out. These effects often determine the reaction outcome such as yield of the desired product and the reaction selectivity. For catalytic processes, the choice of the reaction conditions at the initial catalyst screening stage may play a decisive role in rendering catalyst families potent or catalytically inactive. Operando modeling approaches are necessary to understand the impact of the vast condition space on the behavior of catalytic systems.^3^, ^7^, ^8^

**Figure 1:**
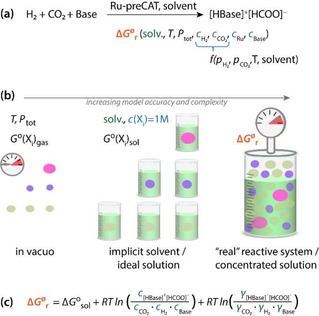
Catalytic CO_2_ hydrogenation to formate salts, the associated fundamental free energy formulations and main conceptual mechanistic assumptions. (a) The homogenous catalytic system for base‐assisted CO_2_ hydrogenation is a multicomponent reactive solution, which thermodynamics and, accordingly, behavior depends on the reaction conditions (e. g. concentrations (*c_i_*), temperature, pressure, nature of solvent, etc.). (b) For obtaining non‐standard free energies (Δ*G* ^ø^_r_) in non‐ideal solutions from the results of DFT calculations, some free energy corrections need to be implemented: standard harmonic approximation (*G*°_gas_), implicit solvation (*G*°_sol_) and concentration corrections for non‐ideality. Although such an approach (c) increased the complexity of considered system, it can be used to analyze the thermodynamics of competing reaction channels in non‐ideal solutions and identify optimal reaction conditions for multicomponent multiphase reactive systems.

To illustrate this, let us consider a representative example of homogeneously catalyzed CO_2_ hydrogenation to formats^9^, ^10^ as a model chemical conversion process. Reductive transformation of carbon dioxide attracts particular attention of the scientific community as it provides a method to store renewable energy in chemical bonds.^11^, ^12^, ^13^ Reversible catalytic CO_2_ hydrogenation^14^ in the liquid phase (Figure 1a) is the key element of a practical reversible H_2_ storage technology.^15^, ^16^ The practical applicability of a catalyst is defined by both its activity and stability under the conditions of the catalytic reaction. The behavior of the complex multiphase catalytic systems common for CO_2_ hydrogenation is defined by both its intrinsic chemistry and the conditions of the practical catalytic process (Figure 1a). In terms of thermodynamics a chemical system aspires to decrease its free energy. Deep understanding of free energy function dependencies allows us to control the chemical reactions and even to reverse deactivation processes. The thermodynamic preference for a particular reaction path (e. g. deactivation or catalytic reaction) in the overall mechanism may depend on such process parameters as solvent, temperature, pressure, concentrations or, more precisely, activity coefficients for all of the components. The fundamental dependencies of the standard‐state reaction Gibbs free energies in solution (Δ*G*°_sol_) on *T*, *P_tot_* and reaction medium can readily be computed using modern electronic structure methods (Figure 1b) by approximating the reactive ensemble with a gas‐phase model, for which the entropic effects are computed from the results of the normal‐mode analysis within the ideal gas approximation at a given *P_tot_* and *T*. When solvent is not participating directly in the chemical transformation, the effect of the medium on the reaction Gibbs free energies may be accounted for with implicit solvent models (e. g. PCM, SMD, etc).^17^, ^18^, ^19^

However, most of these methods, currently widely used in contemporary computational catalysis, do not allow to account for more complex non‐ideal condition‐dependencies such as the variations in the partial pressures (*p_i_*) of gaseous reactants, temperature‐dependence of their solubility and solvent parameters, concentrations as well as non‐ideal activity coefficients reflecting interactions and finite concentrations of the solutes in the reactive system. All these parameters and their interdependencies influence the non‐standard reaction free energies (ΔG^ø^_r_, Figure 1(b)) for the catalytic reaction network involving both the desirable catalytic cycle and various side‐processes leading to the deactivation or degradation of the catalyst.

A comprehensive description of the catalytic system can be achieved with a full kinetic model including information on all intermediates and transitions states for all elementary steps in the reaction network.^7^, ^17^ The construction of such detailed networks using accurate electronic structure methods is a challenge for most practical chemical conversion systems making it extremely difficult to employ full kinetic models for rapid computational screening of potential catalyst systems. Common deactivation paths result in thermodynamically stable states, from which the catalytic cycle cannot be continued.^20^ Therefore, in this work, we propose that although catalysis is a kinetic phenomenon, thermodynamic analysis can be used to probe the deactivation behavior and get an initial assessment of the attainable catalyst lifetime. The concept of condition‐ and concentration‐dependent free energy surfaces^21^ creates a basis for the direct comparison of stabilities of key intermediates under varied conditions, identification of the resting states within the catalytic mechanism and proposing how to avoid their formation by process optimization.

The concept of concentration‐dependent free energies is the basis of the ab initio thermodynamic (aiTD) analysis technique originally introduced by Reuter et al.^24^, ^25^ This method is currently widely used in computational heterogeneous catalysis for the operando modeling of the composition of the reactive surfaces^26^, ^27^, ^28^ and microporous spaces.^29^ Since recently the applicability of this approach has been extended towards analyzing the dynamics of the surface sites under the catalytic conditions.^30^, ^31^ The aiTD method applied to the gas‐solid heterogeneous catalyst systems assumes the ideal gas behavior of the reactive atmosphere to calculate the respective chemical potentials (free energy contributions), while the free energy of the solid surface is approximated with the electronic energy obtained directly from DFT. Formally, the correct free energy description of such heterogeneous catalyst models should also include the account for the fugacity coefficients of the gas‐phase components and the entropic and finite temperature contributions for the solid. However, in practice, these corrections are often assumed to be minor and not affecting the general qualitative trends. These assumptions have been carefully validated in combined experimental and theoretical studies on CO oxidation over Pd surfaces by Rogal et al.^32^ and over Au catalyst by Beret et al.,^33^ in which the composition of the catalyst surface could be adequately described as a function of the partial pressures of the gaseous components.

Such an approximation cannot be directly adopted for the description of liquid solutions or heterogeneous multiphase gas/liquid/solid interfaces commonly encountered in homogeneous catalyst systems. The non‐ideal behavior is expected to manifest itself in the varied solubility of gases, non‐linear condition dependencies of the thermodynamics and activity coefficients. These factors can be accounted for by using the implicit COSMO‐RS solvation model.^34^, ^35^ For example, Green et al employed this approach to investigate the autocatalytic oxidation of hydroxylamine in nitric acid solutions and analyze differences between calculated and experimentally obtained energy functions (Δ*G*, Δ*H*, Δ*S*).^36^

**Figure 2:**
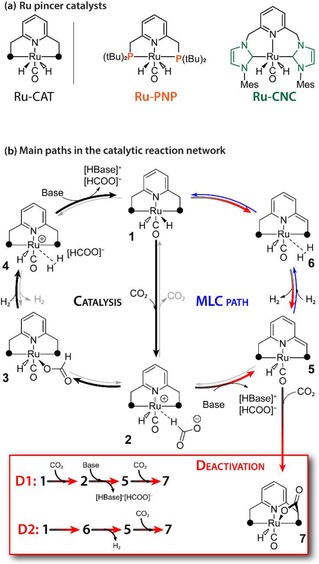
(a) Homogeneous Ru‐PNP^22^ and Ru‐CNC^23^ pincer catalysts active in CO_2_ hydrogenation to formate salts and (b) the main reaction channels including the **Catalysis** 1–2‐3‐4‐1 path and the alternative reaction channels **D1** and **D2** resulting in the long‐term catalyst deactivation.

In this work, we employ a composite DFT‐COSMO‐RS scheme to carry out a rapid and detailed analysis of condition‐dependencies of the thermodynamic parameters in complex multicomponent reactive solutions. As a showcase, we focus on a model homogeneous catalytic reaction that is the base‐promoted CO_2_ reduction to formates catalyzed by homogeneous Ru pincer catalysts (Figure 1a).^22^, ^23^ This catalytic process is perfectly suitable to study the effects of non‐linear condition dependencies on the reaction outcome as, despite its apparent simplicity, the catalytic reaction involves a complex mechanism governed by an extended, yet defined network of elementary reaction steps. Two main paths can be distinguished in this network, namely, the stable catalytic mechanism where the hydrogenation reaction is promoted solely by the Ru center (‘Catalysis’ in Figure 2); and an alternative metal‐ligand cooperative (MLC) path, which may proceed to a thermodynamically‐stable CO_2_ adduct 7 that cannot further promote the catalytic reaction (‘deactivation’). In this study, we consider two Ru catalysts based on an N‐heterocyclic carbene NHC‐based (Ru‐CNC)^23^, ^37^ and phosphine‐based (Ru‐PNP)^22^, ^38^ pincer ligands (Figure 2a). Experimental studies revealed a drastically different deactivation behavior of these catalysts despite similar intrinsic reactivity predicted by DFT calculations.^23^, ^38^

## 2 Results and Discussion

The analysis of the condition‐dependencies was carried out under the mechanistic assumption that the catalytic activity of the Ru pincer catalysts in CO_2_ hydrogenation is defined by the three individual reaction paths outlined in Figure 2. As a starting point for our analysis we first identified the most stable configuration and state of the formate product of the catalytic reaction. Our calculations reveal that within the selected ranges of conditions only the DBU base forms stable adducts (Δ*G*°_r_ <0) with the formic acid (for computed standard energies see Figure S1). The stable form of the [HDBU]^+^[HCOO]^–^ salt in THF is a highly polar contact ion pair that does not form specific interactions with the THF solvent molecules. The formic acid adducts with a weaker Et_3_N base in THF were found to be thermodynamically unstable (Δ*G*°_r_ >0), which is in line with the experimental results that identified this base to be optimal for the reverse reaction of formic acid decomposition to H_2_ and CO_2_.^39^

COSMO‐RS solubility calculations predict the poor solubility of the DBU formate product in THF that is in line with the experimental observations of the rapid product crystallization during the online sampling of the catalytic reaction mixtures. The correct and detailed description of the phase‐separation process in the reactive system is however beyond the capabilities of the current methodology. In the context of the thermodynamic analysis, it is sufficient to only select the representative reference points for the product concentration. Here, we consider only the states of the systems where activities of the base and the product are the same. The base‐formic acid adduct shows a much higher solubility in DMF solvent, which also favorably affect the overall thermodynamics of the reaction as is evidenced by the respective Δ*G*°_r_ values computed for the formation of the [Et_3_NH]^+^[HCOO]^–^ adduct in DMF. However, our preliminary analysis points to very strong solvent‐solute interactions with DMF that could not be adequately accounted for even in the framework of the COSMO‐RS model. Therefore, our further analysis was exclusively focused on the catalytic processes in THF (see Table S2, supporting information).

The computed standard free energies (Δ*G*°) for the competing reaction channels (**Catalysis**, **D1**, **D2**) for Ru‐PNP and Ru‐CNC catalysts suggest that for both systems the DBU/THF solvent combination allow to successfully proceed with the catalytic reaction paths. However, these channels are always in competition with the deactivating paths characterized by the lower Δ*G*° values. Importantly, although for Ru‐CNC both the direct **D1** and base‐assisted **D2** deactivation paths are thermodynamically favorable under the standard conditions, **Catalysis** path is kinetically preferred. The outer‐sphere mechanism of CO_2_ hydrogenation over Ru‐PNP^38^ and Ru‐CNC^23^ proceeds with the overall activation free energy barriers (Δ*G*_act_°) of 67 and 49 kJ/mol, respectively. For Ru‐PNP, standard free energy barriers of 89 and 115 kJ/mol for the **D1** and **D2** paths, respectively, were reported (see the SI).^38^ For Ru‐CNC, the respective Δ*G*_act_° values were 93 and 84 kJ/mol.^23^ This implies that from the kinetic perspective, in the case of Ru‐PNP, the preferred deactivation mechanism is **D1**, whereas the **D2** path is favored kinetically over the Ru‐CNC catalyst. Nevertheless, the **Catalysis** path is always more kinetically favorable and the consideration of the kinetic parameters only would imply the higher stability of the Ru‐CNC than Ru‐PNP, which is in a contrast to the experimental observation. At the common reaction temperatures and under the conditions of standard catalytic tests, the experiments show that the performance of Ru‐CNC is limited due to its rapid deactivation, whereas the Ru‐PNP is catalytically stable. The deactivation is therefore determined by the thermodynamic differences between the kinetically possible reaction channels. Therefore we hypothesize that variations in the condition‐dependencies of the thermodynamics of the competing reaction channels could be used to tune the relative stabilities of the associated key species and therefore alter the stability of the catalytic system.

**Figure 3:**
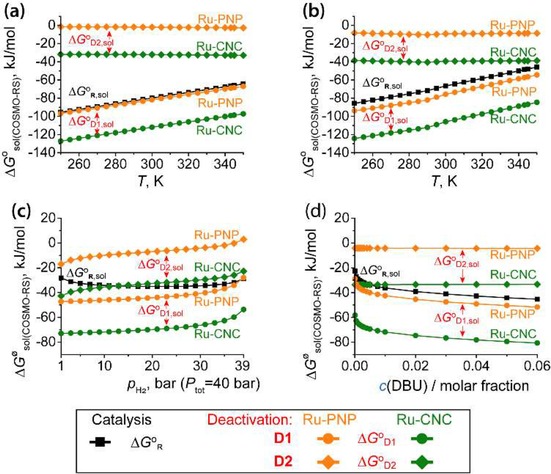
Temperature‐dependencies of free energies of competing elementary processes within the catalytic CO_2_ hydrogenation with Ru pincer catalyst. (a) COSMO‐RS solvent models for an idealized solution reaction (Δ*G*°_sol_) in pure THF, and (b) non‐ideal mixed solvent COSMO‐RS model representing a reaction solution featuring 5 mol% DBU in THF under *p*_H2_=*p*_CO2_=20 bar (*P_tot_*=40 bar). Concentration dependencies of free energies for the competing processes for CO_2_ hydrogenation with Ru pincer catalyst. (c) H_2_ partial pressure dependency (*p*_H2_) of reaction Gibbs free energies at 40 bar total pressure of H_2_:CO_2_ gas mixture and 343.15 K for 5 mol% DBU in THF reactive solution. (d) Reaction Gibbs free energy changes as a function of the molar fraction of DBU in the reactive solution (assumed conditions: *p*H_2_=*p*CO_2_=20 bar, *P_tot_*=40 bar, *T*=343.15 K, THF).

**Temperature** dependencies of free energies is the most common and widely utilized tool to control chemical transformations. These dependencies manifest themselves in both the thermal (enthalpy) and entropic parts of the standard free energy. In non‐ideal solutions, the temperature of the system also affects solubility and activity of the components. COSMO‐RS method, allows us to compute activity coefficients, solubilities and solvation free energies under varied conditions and compositions of the reactive mixtures. For simplicity of the analysis, the temperature dependencies presented in Figure 3(a,b) were computed assuming activities of the main solution components (DBU, [HDBU]^+^[HCOO]^−^, Ru‐catalyst and its derivatives) to be equal to 1. Similarly, activities of CO_2_ and H_2_ in the solution were set to 1 for the calculation of standard free energy changes (Figure 3a). Instead, for the calculation of non‐standard free energy changes with the COSMO‐RS model (Figure 3b), activities of CO_2_ and H_2_ in the solution were directly computed for the system under 40 bar of equimolar H_2_:CO_2_ gaseous mixture with the explicit account for the temperature‐dependencies of the solubilities of the gases. A more detailed discussion on this will be given further in the text.

Temperature dependencies of free energies of reactions within a small temperature range are largely determined by the entropy contribution. These are most pronounced for the reactions that proceed with the change in the number of species such as the **Catalysis** and **D1** paths (Figure 3a,b). With increasing temperatures, both paths become less favored thermodynamically. In turn, particularly for Ru‐CNC, the competing **D2** path becomes effectively promoted.

The effects of non‐idealities and gas‐liquid equilibria under the operando conditions were accounted for in Δ*G* ^ø^_sol_ (Figure 3b) by explicitly considering the solubilities of the gaseous reactants and their respective activity coefficients in the solution. These corrections introduced a minor but pronounced non‐uniform shift of the Δ*G*°_sol_ values for the different reaction channels. The deviation from linear trend in Δ*G* ^ø^_sol_ values at 290–300 K is most likely due to artifacts in COSMO‐RS model parameterization for computing solubility and activity coefficients of the gaseous reactants.

To summarize, Figure 3(a,b) shows that the general trend in the temperature dependency of free energy changes for individual reaction channels does not depend on the environmental model assumed in the calculations. Although the solvent effects and non‐idealities of the system have profound qualitative and quantitative impact on the relative energetics of the competing reaction channels, for the current gas‐liquid multicomponent reaction mixture, the temperature‐dependencies of the free energies are dominated by entropic factors due to the gaseous reactants. As a result, the free energy changes of the competing paths do not cross each other over the whole reaction temperature domain suggesting that the temperature‐dependencies cannot be used to overcome the fundamental tendency of the current system towards long‐term catalyst deactivation under the conditions of catalytic CO_2_ hydrogenation.

**Pressure** of the gaseous reactants provide another gear to tailor the behavior of the current catalyst system. Basic thermochemistry considerations suggest that the preference towards the catalytic cycle or the deactivation channels can be adjusted by varying partial pressures of the reacting gases and, accordingly, their activities in the solution. Therefore, we next analyzed the effect of the change of the molar composition of the CO_2_:H_2_ gas mixture at P_*tot*_=40 bar above the reactive solution on the thermodynamic parameters of the catalyst system (Figure 3c). The concentrations (*c_i_*) of CO_2_ and H_2_ in the selected condition range follow the Henry law and increase linearly with their partial pressures (*p_i_*). The non‐linear changes of the reaction free energies with varying CO_2_:H_2_ ratio (Figure 3c) are due to the pressure‐dependencies of the activity coefficients. Our simulations show that the deactivation **D2** paths can indeed be suppressed by carrying out the reactions under H_2_‐rich conditions. Indeed, the increase of *p*_H2_ is accompanied by the increase of the Δ*G* ^ø^_sol_ for deactivation channels, while the **Catalysis** path is affected to a lesser extent. This allows for a situation when the desirable catalytic path becomes thermodynamically favored over the competing deactivation channels. In particular, for the Ru‐CNC catalyst, the **D2** path is less favorable than the catalytic reaction at H_2_:CO_2_ above ca. 15, while for the Ru‐PNP catalyst **D2** deactivation could be reversed under high hydrogen pressures. For both catalysts, the base‐assisted **D1** channel is the thermodynamically favorable path in a wide range of conditions. Δ*G* ^ø^_r_<Δ*G* ^ø^_**D1**_ is achievable for Ru‐PNP at very high H_2_:CO_2_ ratios. Here we should note that the changes in *p*_H2_ at a constant total pressure of H_2_:CO_2_ influence the behavior of the system indirectly. The key impact is not due to the increase in *p*_H2_ but rather the decreasing *p*_CO2_ that, in turn, affects the CO_2_ solubility with almost no impact on that of H_2_ in the reaction medium. Consideration of solubilities and activity coefficients as Gibbs free energy parameters shown in Figure 3 instead of partial pressures of gases allows us to understand deeper the obtained partial pressure dependencies and rationalize the observed strong *p*_H2_ dependency of the **D1** path.

**Concentrations** of all components of the catalytic mixture impact the thermodynamics of the reactive system. For concentrated solutions commonly encountered in practical homogenous catalysis, the variations of the composition of the liquid phase influence not only the individual activities via concentration dependencies, but also the bulk solvation properties of the medium that can be viewed as a mixed solvent. In the current context, it is most convenient to use the molar fraction of solutes to define concentrations. The solutions with a mole fraction of solutes greater than 0.01 can be already viewed concentrated: that is the mutual interaction between molecules of the solvent and the solutes is not negligible. Experimental studies on CO_2_ reduction with Ru pincers employed reaction mixtures with the molar fraction of the base promotor/co‐reactant as high as 0.06.^38^ Such a high promotor concentration does not allow us anymore to consider the catalytic reaction to take place in a pure THF solvent and a more adequate representation of the reaction medium is a mixed solvent (THF‐DBU) model. By considering DBU as a co‐solvent, the current approach allowed us to simultaneously account for concentration dependencies of solvation energies and the individual activity coefficients in the reactive mixture during the calculation of the reaction free energies of the competing reaction channels. The results in Figure 3d show that the thermodynamic parameters of the reactions with the explicit base participation (**D1** and **Catalysis** paths) show a strong and non‐linear dependence on *c*(DBU) as a result of the changes in activity coefficients. The free energy of the base‐free **D2** path depends only slightly on DBU concentration because of the similar polarity of DBU and THF. If polarities of the components of the solution are substantially different, non‐linear dependencies of free energies could be observed even for those steps, which do not involve the solvent molecules explicitly. This has earlier been observed for the catalytic ester hydrogenation with Mn‐PN in the presence of KO^t^Bu base and THF as the main solvent.^40^ Similar to the partial‐pressure dependencies discussed above, the variation of the DBU base concentration allows establishing the reaction conditions, at which the thermodynamic curves of the **Catalysis** and **D2** deactivation of the Ru‐CNC intersect.

Importantly, in the current model, the concentrations of all other components remain unchanged during the reaction because of (1) the assumption of the semi‐batch operation under constant *p*_H2_ and *p*_CO2_ and (2) very low solubility of the [HCOO]^‐^[HDBU]^+^ product that is predicted to phase‐separate under the catalytic conditions. Therefore, Figure 3d provides direct information into the evolution of non‐standard thermodynamic parameters along the reaction coordinate of CO_2_ hydrogenation.

**Figure 4:**
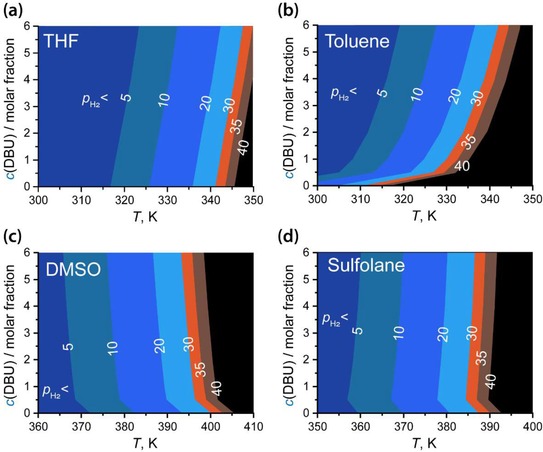
Condition spaces (base concentration, temperature, partial pressure of H_2_) of the preferred Ru‐CNC **Catalysis** path over the **D2** deactivation derived from the respective multidimensional projections of the difference free energy surfaces (ΔΔ*G*) in a) THF, b) toluene, c) DMSO, d) sulfolane solvents. Colored areas in the graph are boarded with contour lines defining points in the condition space at the specified *p*_H2_ (in bars) where ΔΔ*G*=0.

To identify optimal conditions for the catalytic process, the interdependencies between condition parameters have to be accounted for. Because of the non‐additive nature of the individual contributions, the overall free energy function for the system has a strongly non‐linear character. A system approach should be adopted in the thermodynamic analysis by involving explicit consideration of multidimensional condition‐dependencies of the free energy changes. Figure 4 presents the results of such a multidimensional analysis for the Ru‐CNC catalyst system in a form of the (*T*,*p*,*c*)‐projection of relative free energy (ΔΔ*G*) surfaces with the 0 values at given *p*_H2_ shown with the contour lines. In our case we determine ΔΔ*G* as Δ*G* ^ø^_r_–Δ*G* ^ø^_**D2**_. The area to the left of the contour lines correspond to the condition spaces where Δ*G* ^ø^_r_ < Δ*G* ^ø^_**D2**_ making the ΔΔ*G*<0. Here, the catalytic behavior of Ru‐CNC in THF, toluene, DMSO and sulfolane model solvents showing substantially different polarity is compared. Counterintuitively, our analysis shows that *c*(DBU) has a relatively minor impact on the overall process when THF is used as a solvent. In other solvents considered, much more pronounced non‐linear effects of *c*(DBU) on ΔΔ*G* could be seen.

The Le Chatelier's principle and, related, the analysis based on one‐variable functions (Figure 3d) suggests that in THF base concentration could be used as a tool to selectively promote the **Catalysis** path that directly involves DBU as a reagent. The multidimensional analysis reveal that this factor is actually of relatively minor importance and other condition parameters such as reaction temperature and gas phase composition dominate the behavior of the catalyst system. The influence of *p*_H2_ on ΔΔ*G* is limited by the gas solubility. Nevertheless, this parameter is most useful to achieve ΔΔ*G*<0 at elevated temperatures necessary for the optimal reaction kinetics.

Figure 4a shows that at low temperature the desirable CO_2_ reduction is favored at lower *p*_H2_ and therefore the process can potentially be carried out at lower total pressure (*P_tot_*) or in less H_2_‐rich atmosphere. However, the kinetic factors, which are not considered here, will favor the higher‐temperature operation (*T*>340 K),^32^, ^33^ at which the sustained catalytic process necessitates very high *p*_H2_, where the impact of *c*(DBU) is no longer minor. Our computational analysis suggests that by carrying out the catalytic hydrogenation of CO_2_ with Ru‐CNC catalysts at elevated temperature of ca. 353–373 K in excess of H_2_ and DBU base both optimal kinetics and favorable thermodynamics for the catalytic reaction can be achieved resulting in both high rates and stability of the catalytic reaction. The concentration of base is an important promoting parameter when the reaction is carried out in apolar solvents (e. g. toluene, Figure 4b). More polar solvents (DMSO, sulfolane) diminish effectively the promoting effect of the base while increasing the temperature ranges suitable for the catalytic hydrogenation of CO_2_ with Ru‐CNC.

## 3 Conclusion

Summarizing, the presented computational strategy allows reducing the highly complex and challenging kinetic problem of catalyst design to a much more traceable thermodynamic analysis when targeting long‐term stability and high TONs for a catalyst system. The combination of COSMO‐RS method with DFT calculations on stable intermediates within the catalytic reaction network provides a thermodynamic description of the complex multicomponent liquid‐phase catalytic system, and allows operando modeling of the behavior of the overall system under variable conditions. The validity of the approach has been demonstrated on an example of CO_2_ hydrogenation to formates with homogeneous Ru PNP and CNC pincer catalysts, which exhibit drastically different behavior in the catalytic process. Despite having similar reactivity towards CO_2_ hydrogenation, Ru‐CNC complex is much less stable and tends to deactivate via secondary paths under the catalytic conditions. The analysis of condition‐dependent Gibbs free energies for the competing reaction channels within our operando modeling strategy point to favorable condition ranges, in which the deactivation paths are no longer thermodynamically preferred. Operando modeling of catalytic processes in the liquid phase requires the use of non‐ideal mixed‐solvent models accounting for their multicomponent nature and concentration effects. The activities of all components in the reactive solution are the function of both the external conditions (*T*, *P*) and reaction medium composition (*c_i_*, solvent), which directly impact the chemical potentials and the thermodynamics of the associated chemical processes. We propose that the analysis of the multidimensional condition‐dependent free energy surfaces for complex catalytic transformations can be greatly facilitated by considering only the ΔΔ*G* projections of the proposed catalytic and deactivation paths. Importantly, this approach requires an insight about the deactivation and inhibition reaction channels, whereas the details of the catalytic cycle itself are much less important. Our methodology provides a powerful tool to screen the condition space for multicomponent reactions in solution with high efficiency and at a low computational cost, and it can be used to rationally select starting parameters for experimental catalyst tests and activity screenings.

## Experimental Section

In this work, we limited ourselves to considering the thermodynamic dependencies of competing reactions and focused on the analysis of the effect of environmental conditions under the assumption of a sufficient computational accuracy of the selected methodologies for the description of the intrinsic chemistry of the selected catalytic system. Electronic energies of isolated molecular species were computed in the framework of density functional theory using the ORCA (version 4.0.1) program.^41^ Calculations were carried out using the meta‐GGA TPSS^42^ exchange‐correlation functional with the D3 dispersion correction^43^ and valence triple‐zeta polarization Karlsruhe basis set def2‐TZVP for all atoms.^44^ The starting configurations for the catalytic intermediates were adopted from refs.^23^, ^38^ and optimized using the current computational procedure. The advanced sampling techniques in combination with an explicit solvent model allow for the direct and accurate calculation of thermodynamic parameters for the given chemical composition of the reactive system under defined conditions. However, the associated computational methods are too demanding for the screening of extended condition spaces when applied to practical chemical systems. In this work, bulk solvent effects have been accounted for by using implicit solvation models, namely, the C‐PCM,^45^ SMD,^46^ and COSMO‐RS.^34^, ^35^ To correctly describe the contact ion pair [HBase]^+^[HCOO]^−^, the C‐PCM approach was applied during geometry optimization. COSMO‐RS calculations were carried with the CosmoTherm (version 16)^34^, ^47^ and Turbomol (version 7.2)^48^ programs using the recommended set of parameters (BP_TZVPD_FINE_C30_1601).

The reaction free energies were calculated according to the equations shown in Figure 1. Free energies of gas phase structures were obtained with quasi rigid‐rotor‐harmonic‐oscillator (QRRHO) approximation.^49^ Thermodynamic dependences were simulated within the following boundary conditions: *T*=250–350 K, *P_tot_*=40 bar, *c*(DBU)=0.01–6 mol %, for which sufficient experimental references are available. Here we are limited by borders of the existence of liquid systems, where the solvent model approximation seems adequate. It may be noted that we are also confined by accuracy of used models: transition states calculations could contain substantial error, strong interactions with solvent species are not considered. Standard free energies were calculated in the gas phase and then transformed into the desired temperature conditions within the QRRHO approach. The solvation free energies were referred to the 1 mol gas/1 mol solvent and were calculated for each value of the temperature, partial pressure and base concentration. The solubility of gaseous reactants and their activity coefficients were calculated for each temperature, partial pressure and base concentration. Activities of the base were calculated in accordance with set concentration and modeled activity coefficients.

## Conflict of interest

The authors declare no conflict of interest.

## Supporting information

As a service to our authors and readers, this journal provides supporting information supplied by the authors. Such materials are peer reviewed and may be re‐organized for online delivery, but are not copy‐edited or typeset. Technical support issues arising from supporting information (other than missing files) should be addressed to the authors.

Supplementary
