# Supplementary material for: Operando Modeling of Multicomponent Reactive Solutions in Homogeneous Catalysis: from Non‐standard Free Energies to Reaction Network Control
Source: ChemCatChem. 2019 Dec 11;12(3):795–802. doi: 10.1002/cctc.201901911 (PMC7043346; doi:10.1002/cctc.201901911)
Supplement: Supplementary file 1 — Supplementary [file CCTC-12-795-s001.pdf]

CHEMCATCHEM

Supporting Information

Operando Modeling of Multicomponent Reactive Solutions in Homogeneous Catalysis: from Non-standard Free Energies to Reaction Network Control

Pavel O. Kuliaev and Evgeny A. Pidko*© 2019 The Authors. Published by Wiley-VCH Verlag GmbH & Co. KGaA. This is an open access article under the terms of the Creative Commons Attribution License, which permits use, distribution and reproduction in any medium, provided the original work is properly cited.

Supporting Information

Operando modeling of multicomponent reactive solutions in homogeneous catalysis: from non-standard free energies to reaction network control

Pavel O. Kuliaev [b] and Evgeny Pidko * [a,b]

[a] TheoMAT Group, ITMO University, Lomonosova 9, St. Petersburg 191002, Russia

[b] Inorganic Systems Engineering Group, Department of Chemical Engineering, Delft University of Technology, Van der Maasweg 9, 2629 HZ Delft, The Netherlands

Corresponding author: Evgeny A. Pidko (e.a.pidko@tudelft.nl)

Contents:

Products optimization 3

Standard free energies of catalytic CO_2 hydrogenation in THF solution 4

Temperature dependencies of free energies in catalytic system...5

Kinetics of catalytic CO_2 hydrogenation reaction 6

References...7

Products optimization

Previous experimental studies revealed that CO_2 hydrogenation with Ru-PNP pincer catalyst in a THF medium proceeds with good yields to DBU formate.[S1] However, the initial calculations utilizing previously reported geometries of DBU formate salts and gas-phase optimized geometries delivered positive values of standard free energies for such a catalytic process (**Figure S1**b,c). We have identified that this apparent inaccuracy is mainly due to the errors encountered during the calculation of the energy of the contact ion pair ion pair in THF. Attempts to find a stable adduct with solvent molecules (as in the case of DMF, **Table S1**) were also in vain. A detailed study of the simulation results revealed an insufficient charge distribution on the components of the ion pair that results from the gas-phase approximation employed. When the geometry optimization was carried out using the PCM model, a more favorable geometry of the DBU formate product was obtained (**Figure S1**a). Such polar structure of salt gives lowest solvation energy in polar solvents, while an existence of simple ions seems impossible. The energy differences between the salt structures are shown in **Table S2**.

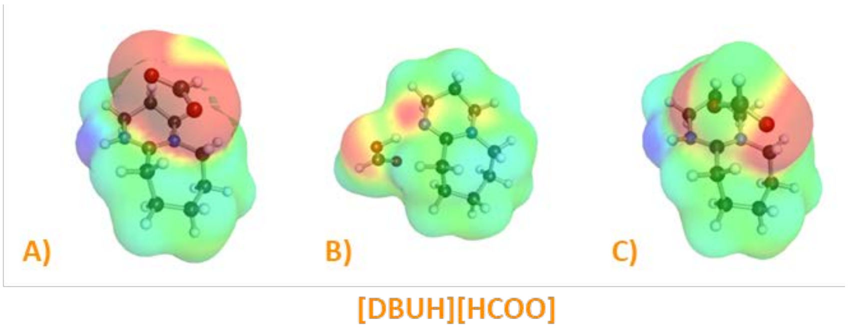

Figure S1. Structures of optimized DBU-formate ionic pairs with cavity shells including local surface charges: positive charge colored in blue, negative charge colored in red. A) PCM optimized; B,C) gas phase optimized.

Experimentally, it was found that the highest reaction rates and CO_2 hydrogenation yields are obtained when using N,N-dimethylformamide (DMF) as a solvent.[S1] COSMO-RS calculations identify a number of favorable products that could be formed in DMF solution, some of which do not represent the DBU formate adducts, but rather take form of adducts with DMF solvent molecules (**Table S1**). Such strong solvent effects cannot be adequately described by the implicit solvent models. On the other hand, the inclusion of explicit solvent molecules in the models of solvated complexes makes the resulting molecular models incompatible with the COSMO-RS solvent model. As shown in **Table S1** the main form of product in DMF might be the formic acid while salt kinds of products seem to be unstable.

Structure of product	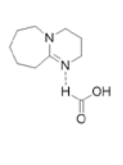	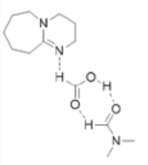	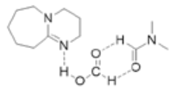	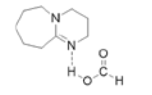	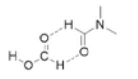	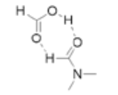	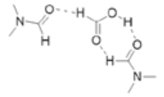
ΔG^0_{gas} (kJ/mol)	20	-52	Not stable	-23	-54	-75	-112
ΔG^0_{DMF} (kJ/mol)	16	4	Not stable	54	-22	-30	-59

Table S1. Standard Gibbs free energies of formation of different potential CO_2 hydrogenation product computed within the gas-phase approximation and using the COSMO-RS model in pure DMF.

Standard free energies of catalytic CO_2 hydrogenation in THF solution

	ΔG^0_{THF} (kJ/mol) for deactivation D1		ΔG^0_{THF} (kJ/mol) for Catalysis			ΔG^0_{THF} (kJ/mol) for deactivation D2	
	**7** Ru-PNP	**7** Ru-CNC	$[HBase][HCOO]$	HCOOH	$[HCOOH]_2$	**7** Ru-PNP	**7** Ru-CNC
DBU	Fig. S1a) -78	Fig. S1a) -97	Fig. S1a) -74<br>Fig. S1b) 28<br>Fig. S1c) 69	24	74	7	-21
Et_3N	50	22	43				

Table S2. Calculated values of standard free energy changes ΔG^0_{solv} for competing / alternative reaction channels in pure THF (COSMO-RS).

The results presented in the **Table S2** suggest that the use of (Et_3N) triethylamine base in the catalytic CO_2 hydrogenation to formates in THF should be very inefficient, which is consistent with the experimental results showing that this base is preferred for the reverse dehydrogenation reaction.[S2] Only one salt product is stable in THF media, the structure is shown in **Figure S1a.** The initial analysis of the computed standard free energies in pure THF (**Table S2**) reveals that DBU base is required to proceed favorably with the catalytic hydrogenation reaction. Standard Gibbs free energies for the deactivation path D1 are comparable to that of the target catalytic process.

Temperature dependencies of free energies in catalytic system

Temperature dependencies of free energies of reactions within a small temperature range are largely determined by the entropy contribution. These are most pronounced for the reactions that proceed with the change in the number of species such as the **Catalysis** and **D1** paths (**Figure S2**). In general, the inclusion of solvent corrections gives rise to more negative free energies for all channels as compared to the gas-phase calculations and, importantly, also affects the relative favorability of the competing reaction paths. The comparison of the data presented in **Figure 3** (in main text) reveals a substantial qualitative difference in the energetics predicted by the C-PCM and COSMO-RS implicit solvent models, respectively. These differences are attributed to inaccuracies in modeling solvation of the ionic compounds [S3] that lead to underestimated solvation energies computed within the C-PCM model. Indeed, the results in **Figure S2b** suggest that rapid deactivation of both Ru-PNP and Ru-CNC pincer catalysts should be inevitable in the presence of H_2:CO_2 mixture. This effectively contradicts the experimental observations of the extremely high stability of Ru-PNP catalyst in the presence of the base under varying atmosphere and temperature (with high activity observed above 343K) of a practical formate-based hydrogen storage device [S2].

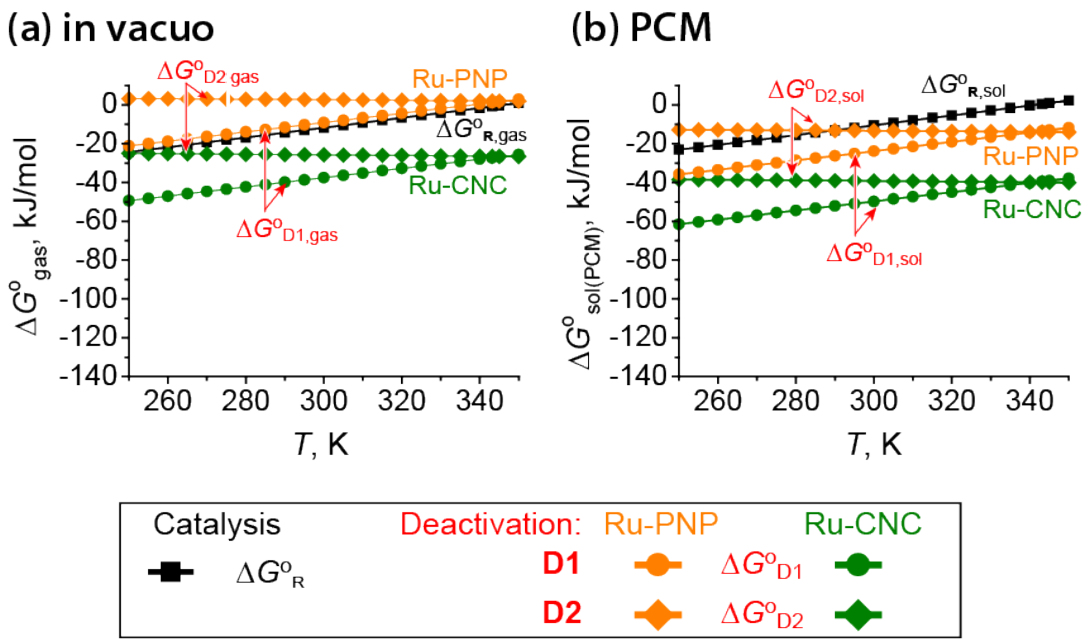

Figure S2. Temperature-dependencies of Gibbs free energies of competing elementary processes within the catalytic CO_2 hydrogenation with Ru pincer catalyst: (a) gas-phase (in vacuo) approximation; (b) implicit C-PCM.

Kinetics of catalytic CO_2 hydrogenation reaction

Previously the considered systems were studied in D3-PBE0/6-311G*/PCM) level of theory[S4,S5]. Despite the smaller operating range of external conditions, interest in the use of Ru-CNC catalyst is due to two important factors: the relative simplicity of synthesis and better kinetic properties (**Figure 3S**).

As shown in Figure S3, the kinetic barriers for D1 deactivations are much bigger than for Catalysis. Thus these mechanisms might be greatly suppressed by kinetics, however, this limitation may vanished with increasing temperature in reactive system. On the other hand D2 mechanism seems more kinetically favorable especially for Ru-CNC catalyst.

Analyzing these results we conclude that under lower temperatures Ru-CNC catalyst deactivates only by D2 mechanism, while under higher temperatures it deactivates by both deactivation mechanisms. Therefore optimization of the thermodynamics of the catalytic process should consider the temperature range where deactivation processes are kinetically suppressed while catalytic reaction is kinetically allowed.

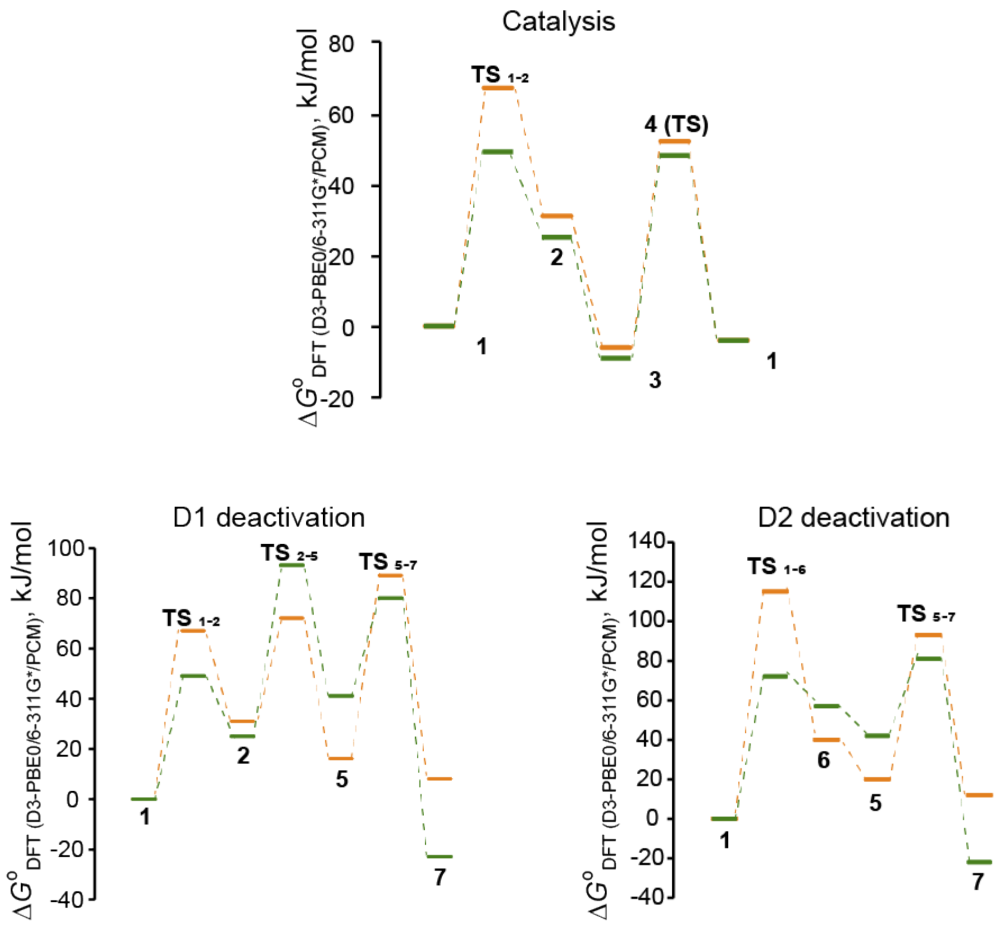

Figure S3. Calculated values of standard free energies of intermediates and transition states obtained in previous works[S4,S5]. Ru-CNC catalyst structures colored in green, Ru-PNP structures colored in red.

References

[S1] Filonenko, G. A., Hensen, E. J. M. & Pidko, E. A. Mechanism of CO_2 hydrogenation to formates by homogeneous Ru-PNP pincer catalyst: from a theoretical description to performance optimization. *Catal. Sci. Technol.* **4**, 3474–3485 (2014).

[S2] Filonenko, G. A., Van Putten, R., Schulpen, E. N., Hensen, E. J. M. & Pidko, E. A. Highly efficient reversible hydrogenation of carbon dioxide to formates using a ruthenium PNP-pincer catalyst. *ChemCatChem* **6**, 1526–1530 (2014).

[S3] Wang C, Ren P, Luo R. Ionic Solution: What Goes Right and Wrong with Continuum Solvation Modeling. *J Phys Chem B* **121**, 11169-11179 (2017).

[S4] Filonenko GA, Conley MP, Copéret C, Lutz M, Hensen EJM, Pidko EA. The impact of Metal–Ligand Cooperation in Hydrogenation of Carbon Dioxide Catalyzed by Ruthenium PNP Pincer. *ACS Catal* **3**, 2522-2526 (2013).

[S5] Filonenko GA, *et al.* Catalytic Hydrogenation of CO2 to Formates by a Lutidine-Derived Ru-CNC Pincer Complex: Theoretical Insight into the Unrealized Potential. *ACS Catal* **5**, 1145-1154 (2015).
